# Inhomogeneous Strain Behaviors of the High Strength Pipeline Girth Weld under Longitudinal Loading

**DOI:** 10.3390/ma17122855

**Published:** 2024-06-11

**Authors:** Zhihao Zhang, Yan Ma, Shuo Liu, Lihong Su, Leigh Fletcher, Huijun Li, Baosen Wang, Hongtao Zhu

**Affiliations:** 1 School of Mechanical, Materials, Mechatronic and Biomedical Engineering, Faculty of Engineering & Information Sciences, University of Wollongong, Wollongong, NSW 2522, Australia; zz215@uowmail.edu.au (Z.Z.); yanm@uow.edu.au (Y.M.); lihongsu@uow.edu.au (L.S.); a.leigh.fletcher@gmail.com (L.F.); huijun@uow.edu.au (H.L.); 2 Baosteel Research Institute, Baoshan Iron & Steel Co., Ltd., Shanghai 201900, China; liushuo@baosteel.com (S.L.); bswang@baosteel.com (B.W.)

**Keywords:** pipeline girth weld, cross-weld tensile test, digital image correlation, reinforcement, strain migration

## Abstract

Unforeseen failures in girth welds present a significant challenge for the pipeline industry. This study utilizes 3D Digital Image Correlation (DIC) assisted cross-weld tensile testing to analyze the strain response of high-strength thick-walled pipelines, providing essential insights into the strain migration and fracture mechanisms specific to girth welds. The results reveal that the welding process significantly affects the mechanical distribution within the girth weld. The tested Shielded Metal Arc Welded (SMAW-ed) pipe exhibited undermatched girth welds due to high heat input, while Gas Metal Arc Welding (GMAW) introduced a narrower weld and Heat-Affected Zone (HAZ) with higher hardness than the base metal, indicative of overmatched girth welds. Strain migration, resulting from a combination of metallurgical heterogeneous materials and geometrical reinforcement strengthening, progressed from the softer HAZ to the base metal in the SMAW-ed sample with reinforcement, ultimately leading to fracture in the base metal. In contrast, the GMAW-ed sample shows no strain migration. Reinforcement significantly improves the tensile strength of girth welds and effectively prevents failure in the weld region. Sufficient reinforcement is crucial for minimizing the risk of failure in critical areas such as the weld metal and HAZ, particularly in SMAW-ed pipes.

## 1. Introduction

Recently, the pipeline industry has faced significant challenges due to unforeseen failures in girth welds. In the past four years, at least 30 incidents have been reported in which girth welds have failed [1]. The studies by Wang et al. [2,3] highlight that these failures have affected a range of pipelines, including those newly constructed, those in service for a few years, and even those not yet operational. Despite the prevalence of these issues, a comprehensive and detailed examination of the inhomogeneous strain behaviors of girth welds has yet to be conducted.

The integrity of pipeline girth welds is dependent on the welding process utilized. Currently, the industry predominantly employs SMAW and GMAW for joining pipeline steel. SMAW, a traditional manual welding technique, does not require flux or shielding gas, rendering the equipment straightforward to use outdoors and easily transportable [4]. The complexity of real-world welding scenarios often necessitates manual operation. Compared to SMAW, GMAW involves semi-automatic or fully automatic equipment, offering consistent stability that typically leads to uniform welds with fewer defects [5]. This process usually produces a narrower weld and HAZ, as well as lower heat input and reduced HAZ softening [6,7,8,9]. Therefore, GMAW weld joints typically have better mechanical properties. Understanding the impact of various welding processes on girth weld failures is essential, yet, as of now, there are no detailed reports addressing this issue.

Although the welding techniques employed for pipeline girth welds are well-established, areas for enhancement remain, particularly concerning the specification for weld reinforcement. Effective control of the reinforcement can significantly improve the quality of the weld bead, subsequently bolstering the mechanical properties of both the weld and HAZ. Wang et al. [10] and Ma et al. [11] found that appropriate reinforcement not only increased the ultimate tensile strength and elongation of samples but also helped in redistributing stress to minimize failures. The challenge in systematically studying weld reinforcement arises from the non-uniformity of materials among base metal, weld, and HAZ, as well as the complex geometry of welds. In the context of Australian oil and gas pipeline standards, AS 2885.2 [12] serves as the primary guideline for pipeline design and installation, covering various inspection and evaluation criteria for pipeline girth welds. However, this standard does not include specific regulations for reinforcement. This omission suggests that, in certain extreme scenarios, a girth weld with no reinforcement (zero reinforcement height) might still comply with current regulations. Considering the acknowledged positive impact of reinforcement on the mechanical properties of girth welds, a targeted study on reinforcement could provide valuable insights and contribute to refining evaluation criteria for pipeline girth welds. This is particularly crucial given the challenges posed by unexpected failures in these welds for the pipeline industry.

Internal pressure in pipeline service conditions significantly impacts the deformation behavior of both the pipeline steel and the girth welds. Using finite element analysis, Yang et al. [13] found that internal pressure greatly affects the strain capacity of undermatched girth welds. Specifically, when internal pressure increases from 0 MPa to 10 MPa, the strain capacity of the pipeline decreases by approximately 81%. Igi et al. [14] examined the effect of internal pressure on the tensile strain capacity of pipelines, finding that high internal pressure increases the crack-driving force of surface notches and significantly reduces the critical tensile strain. In addition to introduce solid element in modeling pipe deformation, Fonseca et al. [15] developed a trigonometric function to use tubular ring element to measure the transverse displacement of the pipe.

Digital Image Correlation (DIC) is a powerful non-contact optical measurement technique utilized to evaluate the deformation and displacement characteristics of various materials and structures. One of DIC’s key advantages is its capability to capture full-field deformation, which encompasses the entire surface of a specimen [16,17,18,19,20,21]. This feature is particularly beneficial for structures such as pipeline girth welds, which exhibit nonuniform material properties and inhomogeneous deformation behavior. By integrating DIC with tensile testing, Peng et al. [22] successfully determined the local constitutive properties of welded steel joints. This approach enabled a more detailed understanding of the material properties of welds and HAZ. Huda et al. [23] employed DIC to distinguish the variations between local and global tensile behaviors across different sections of a submerged arc-welded specimen. This study offered a thorough analysis of how different areas of the specimen responded under tension. Furthermore, the research by Zhang et al. [24] provided a comprehensive understanding of the tensile strain evolution in girth welds during the entire tensile testing process. This highlighted the utility of DIC in capturing dynamic strain variations, thereby offering crucial insights into the behavior of welded structures under stress. Although the DIC technique is commonly employed to measure the tensile deformation of pipe steel, there are only limited studies examining its strain evolution in different tensile stages for inhomogeneous materials like cross-weld specimens. In particular, there is a lack of detailed analysis regarding the migration of maximum tensile strain among different regions of the weld structure, specifically between the weld, HAZ, and the base metal.

Understanding the root causes of girth weld failures is crucial for preventing their occurrence in the future. The current study, employing DIC to analyze the complete tensile response of high-strength pipelines, offers critical insights into the fracture mechanisms specific to girth welds. The intriguing phenomenon of maximum tensile strain migration among different regions of the weld structure, particularly between the weld, HAZ, and base metal, was investigated, and a corresponding mechanism was proposed. Comprehending these mechanisms is essential for understanding the dynamics of strain response in welded joints, as they can significantly affect their integrity and performance. Furthermore, current research explored the influence of different welding processes and reinforcements on these failures. The findings of this research are valuable not only for understanding the inhomogeneous strain behaviors of high-strength pipeline girth welds under longitudinal load but also for the development and refinement of industry standards concerning pipeline girth welds. They contribute to the development of safer and more reliable pipelines.

## 2. Experimental Procedure

### 2.1. Materials

X65 pipeline steel is widely used for oil and gas transportation due to its favorable mechanical properties. This study focuses on two high-strength thick-walled API 5L X65 pipes, each welded using different methods: SMAW and GMAW, respectively. Both pipes were coated with Fusion Bonded Epoxy (FBE), a common corrosion protection method in the pipeline industry. Consequently, they were in the strain aged condition prior to sampling. The yield strength of the pipes increased by 30–50 MPa after coating, while the ultimate tensile strength remained constant [25]. For clarity, the pipelines are designated as P1 and P2 throughout the study. The chemical composition of both pipeline steels is presented in Table 1.

Acknowledging the significance of welding procedure qualification testing, it was essential to fully replicate service conditions, including any heat treatment effects induced during coating. Therefore, sample sectioning was conducted through cold machining to ensure the precision and reliability of the current study’s findings. accurately reflecting the mechanical properties of the in-service pipelines.

Table 2 outlines the specific welding processes and parameters employed. For pipe P1, with a wall thickness of 17.5 mm, the initial root pass was executed using GMAW, followed by the fill and cap passes completed with SMAW. In contrast, pipe P2, with a slightly thicker wall at 19.8 mm, had its girth welds entirely produced using GMAW. The welding consumables used for the fill and cap passes varied between pipes P1 and P2, as shown in Table 2. It is important to highlight that the heat input of the fill and cap passes for P2, utilizing the GMAW welding process, was significantly lower than that of P1, which was welded using the SMAW technique.

### 2.2. DIC-Assisted Cross-Weld Tensile Test

**Figure 1:**
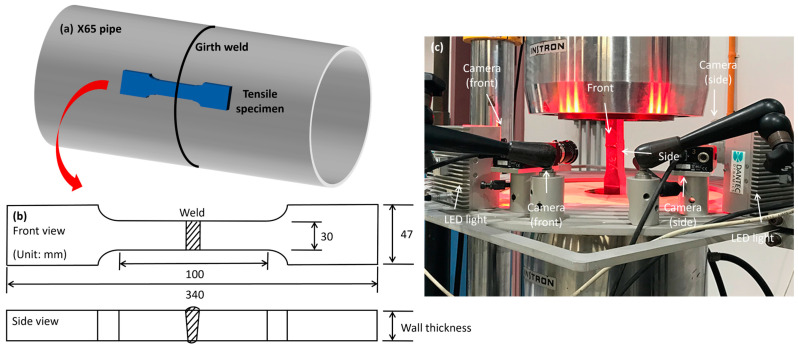
(**a**) Sample sectioning position in the pipe; (**b**) Cross-weld tensile sample; (**c**) Equipment of cross-weld tensile test and DIC system.

The specimens, sectioned from a girth welded X65 pipeline, were manufactured for the cross-weld tensile tests in accordance with the AS 2205.2 testing standards [26]. As shown in Figure 1a, the specimens were cut from the girth-welded pipes with the weld bead positioned at the center. The reduced section, as depicted in Figure 1b, measures 100 mm in length and 30 mm in width, with the sample thickness matching that of the pipe wall. To accurately assess the impact of reinforcement on tensile failure, additional samples were prepared by removing the weld cap and root from the standard tensile specimens, thus producing samples without reinforcement.

The tensile tests were performed using an Instron 8033 testing machine at a constant strain rate of 1 × 10^−3^ s^−1^ [27]. The tests in each condition were conducted three times to guarantee repeatability. Traditional cross-weld testing typically measured only the fracture load and the location of the fracture whether in the weld metal, base metal, or HAZ. In the current study, the DIC technique was employed to further capture the strain distribution across the specimen and track maximum strain migration among inhomogeneous welds, HAZ, and the base metal during the tensile test. Previous research primarily utilized DIC cameras to observe 2D deformation from either the front view or side view of the sample, often neglecting the other aspect [22,23,28]. As shown in Figure 1c, in our research, we introduced four high-resolution cameras with a high resolution of 1172 × 2050 pixels, two aimed at the front and two capturing the sides. The images were recorded every half second. The front cameras focused on the strain behavior across the reduced section region, while the side cameras simultaneously monitored the strain distribution within the girth weld region. This new setup, incorporating both front and side views, allowed for comprehensive recording of the 3-dimensional full-field deformation of the girth weld, enhancing the accuracy in pinpointing strain concentrations. This, in turn, facilitated a more precise analysis of the mechanisms behind maximum strain migration and why the fracture did not occur at a relatively lower hardness region. Illumination was provided by the LED lights. To ensure clear camera identification of all information, the entire surfaces of the specimen were prepared by spraying black and white paints to create a random speckle pattern for strain measurement.

### 2.3. Microstructure Characterization and Hardness Mapping

The microstructures of the weld metal (WM), HAZ, and base metal (BM) are significantly different and play a critical role in the inhomogeneous strain behaviors observed in girth-welded pipes. Specimens were sectioned from the original pipe to represent these varied regions for detailed microstructural characterization. After being polished and etched with a 2% nital solution, the samples were examined using a Nikon LV100NDA optical microscope (Tokyo, Japan).

Hardness mapping is crucial in classifying the heterogeneous weld properties and the resulting variable mechanical characteristics among the inhomogeneous materials of the weld metal, HAZ, and base metal. Using a Via-F automatic hardness tester, one kgf Vickers hardness maps were produced from the polished cross-sections of the girth weld specimens. The scanning area was meticulously selected to encompass the weld, HAZ, and base metal, with a step size of 0.5 mm and approximately 2200 total indentation points, following the ISO 6507-1:2018 standard [29].

## 3. Results and Discussion

### 3.1. Heterogeneous Microstructures of Base Metal, Weld Metal and HAZ

**Figure 2:**
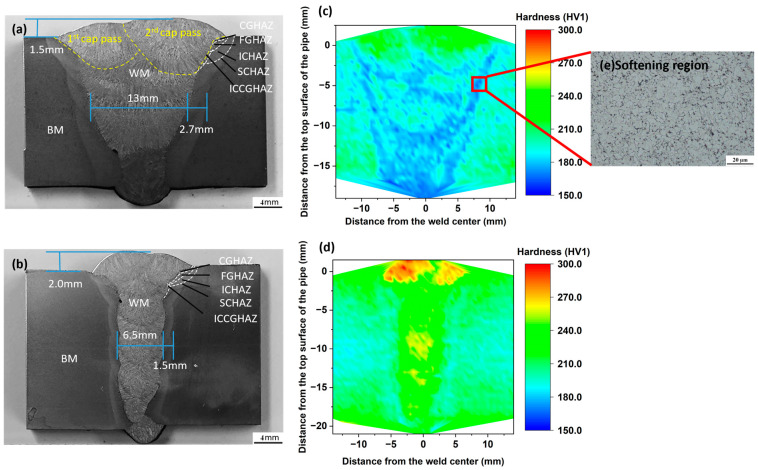
Geometry and morphology of cross-weld sections of (**a**) SMAW-ed P1 and (**b**) GMAW-ed P2; Hardness mapping of (**c**) P1 and (**d**) P2 girth weld; (**e**) Microstructure of the softening region.

The geometry and macro-morphology of cross-weld specimens P1 and P2 are illustrated in Figure 2a and Figure 2b, respectively. Both specimens comprise three distinct materials, including base metal, weld metal, and HAZ. The weld metal is produced through root welding passes and multiple fill and cap welding passes. The differentiation between these passes is noticeable by subtle changes in the orientation and distribution of the weld microstructure, which are further highlighted by corresponding variations in color and shape within the HAZ. It is important to note that the caps of both P1 and P2 are formed by two weld passes, resulting in intersecting top profiles. This contributes to the inhomogeneous strain distribution, which is discussed further in Section 3.3.

The microstructure of the HAZ is more complex due to the multiple weld passes and heat dissipation into the base metal. The HAZ refers to the area of base metal adjacent to the weld but not melted during the welding process. As depicted in Figure 2a,b, according to the peak temperature and cooling time during welding, the HAZ can be divided into several sub-regions, including the coarse-grained heat-affected zone (CGHAZ), fine-grained heat-affected zone (FGHAZ), inter-critical heat-affected zone (ICHAZ), and subcritical heat-affected zone (SCHAZ) [30,31]. An interesting sub-region, the inter-critical reheated coarse-grained heat-affected zone (ICCGHAZ), is formed due to the tempering of the preformed CGHAZ by subsequent welding passes [32,33], which results in a structure that differs from the typical CGHAZ.

The geometry of the weld and HAZ in these two girth welds correlates with the heat input from different welding practices. As shown in Figure 2a,b, the central lines of the weld metals for SMAW-ed P1 and GMAW-ed P2 measure 13 mm and 6.5 mm in width, respectively, indicating a broader weld for SMAW-ed P1 and a narrower one for GMAW-ed P2. Additionally, the average width of the HAZ for the P1 sample is 2.7 mm, which is considerably larger than the 1.5 mm for the P2 sample. Furthermore, the average height of the reinforcement of SMAW-ed P1 and GMAW-ed P2 are 1.5 mm and 2.0 mm, respectively, which are 8.5% and 10% of the base metal thickness respectively.

**Figure 3:**
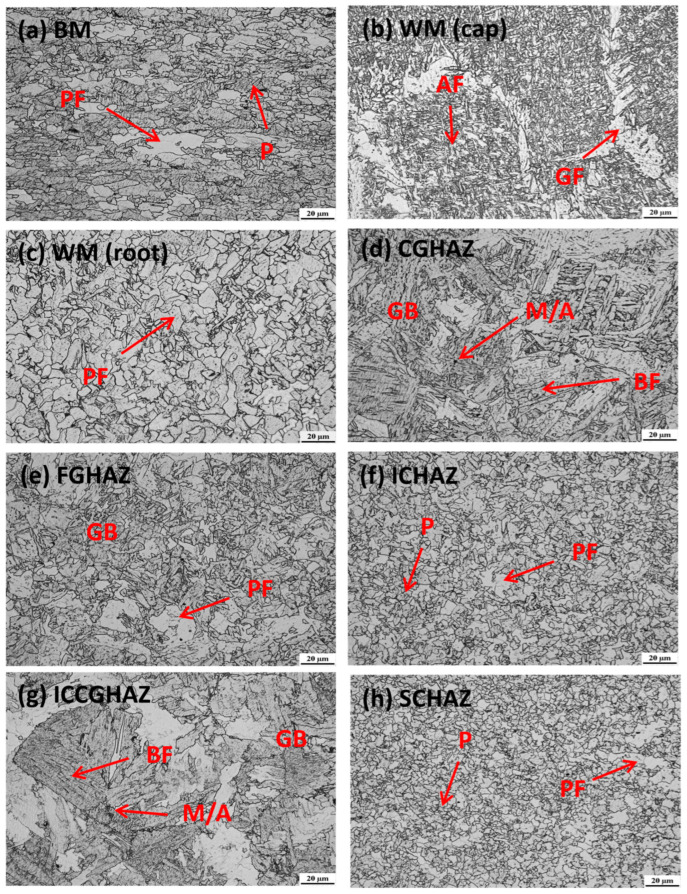
Microstructure of the SMAW-ed P1 girth weld. P: pearlite, PF: polygonal ferrite, AF: acicular ferrite, GF: grain boundary ferrite, BF: bainitic ferrite, GB: granular bainite, and M/A: martensite–austenite constituent.

**Figure 4:**
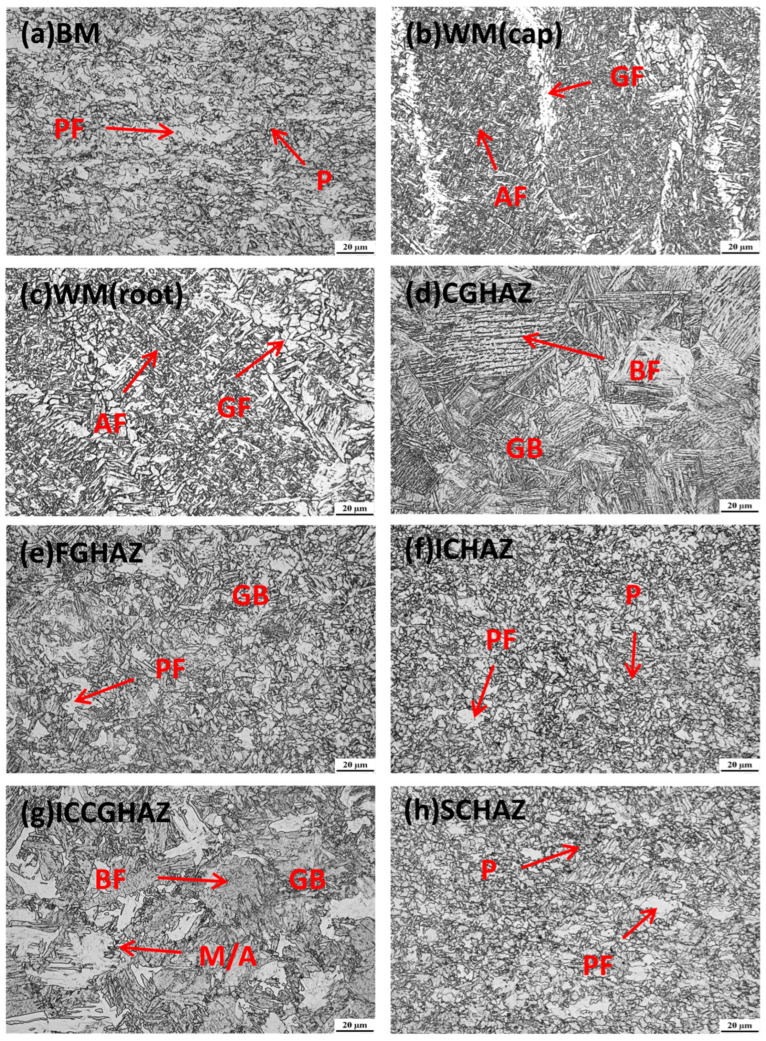
Microstructure of the GMAW-ed P2 girth weld. P: pearlite, PF: polygonal ferrite, AF: acicular ferrite, GF: grain boundary ferrite, BF: bainitic ferrite, GB: granular bainite, and M/A: martensite–austenite constituent.

Figure 3 and Figure 4 show the detailed heterogeneous microstructures of BM, WM and HAZ. As evidenced by Figure 3a and Figure 4a, there is no notable microstructural difference between the base metal of samples P1 and P2, which are primarily composed of polygonal ferrite (PF) and islands of pearlite (P). This similarity in microstructure corresponds to the nearly identical chemical compositions of the two samples, as presented in Table 1.

Due to differences in welding consumables and welding process, the microstructures of weld metal in the two samples exhibit significant variations. For the SMAW-ed P1 specimen, the microstructure of the weld cap pass, depicted in Figure 3b, mainly consists of grain boundary ferrite (GF) and acicular ferrite (AF). The root pass is subjected to a lower heat input compared to fill & cap passes, resulting in the formation of fine polygonal ferrite as shown in Figure 3c. In contrast, for the GMAW-ed P2, both cap pass and root pass are composed of grain boundary ferrite and acicular ferrite, while the grain size in these areas is smaller than that of the SMAW-ed P1 cap pass, as shown in Figure 4b,c. The heat input of GMAW is lower than that of SMAW, which leads to finer grain in GMAW. These grains encounter difficulties in growth after nucleation due to the reduced heat input [34,35].

The microstructures of HAZ subregions of the P1 and P2 specimens are displayed in Figure 3d–h and Figure 4d–h. The CGHAZs of both samples mainly consist of bainitic ferrite (BF) and granular bainite (GB), as shown in Figure 3d and Figure 4d. However, there are morphological differences in the CGHAZs of P1 and P2. As shown in Table 2, the weld joint of the SMAW-ed P1 sample experiences higher heat input, resulting in a coarsening of ferrite and an increased proportion of granular bainite. Meanwhile, a small amount of martensite-austenite (M/A) components can be observed in the CGHAZ of P1 in Figure 3d. For the GMAW-ed P2 sample, the bainite is distributed as fine laths, with visible original austenite grains. The FGHAZ in Figure 3e and Figure 4e is mostly composed of polygonal ferrite and granular bainite. P2 exhibits a finer microstructure than P1 due to lower heat input. The ICHAZ and SCHAZ of both specimens are similar to the base metal, consisting of polygonal ferrite and a small amount of pearlite, as shown in Figure 3f,g and Figure 4f,g. The ICCGHAZ in Figure 3h and Figure 4h has a microstructure similar to the CGHAZ, consisting mainly of bainitic ferrite, granular bainite, and M/A. However, more M/A are precipitated at the grain boundary, which greatly reduces toughness and increases the risk of brittle fracture [36,37].

### 3.2. Heterogeneous Mechanical Properties of Girth Welds

Reflecting the non-uniform microstructural properties of the base metal, weld metal and HAZ, the mechanical characteristics of the inhomogeneous girth weld also vary significantly. The hardness mapping of the cross-weld section of the SMAW-ed P1, as shown in Figure 2c, reveals that the base metal has a hardness value ranging from 190 to 210 HV. The cap pass of the weld metal exhibits a hardness of around 230 HV, which is higher than that of the base metal, while the fill and root pass has a hardness of approximately 170 HV. Clear softening areas are observed in the HAZ and at the interfaces between the weld passes. The average hardness of these softening regions is approximately 20 HV lower than that of the base metal. The microstructure of the softened HAZ is the typical ICHAZ microstructure, as shown in Figure 2e. In these regions, although the temperature is not high enough to cause significant grain growth, the material undergoes slight annealing or tempering, leading to the coarsening and softening of carbides, consequently reducing the material hardness. The slower cooling rate associated with SMAW can further exacerbate these effects, resulting in reduced hardness and strength in the HAZ and at the interfaces between the weld passes compared to the base metal.

The hardness mapping of the GMAW-ed P2 girth weld is presented in Figure 2d. The results show that the hardness of the weld metal is higher than that of the base metal, with a value of approximately 250 HV compared to around 200 HV for the base metal. The use of the same welding consumable and welding process for both cap and root passes results in similar hardness levels throughout the weld region, with slight hardening observed on the top of the weld, with a value of approximately 280 HV. The hardness of the HAZ is identical to that of the base metal, without any clear HAZ softening. GMAW involves lower heat input and faster cooling rates compared to SMAW. These conditions minimize the thermal impact on the material, thus maintaining the relatively high mechanical properties of the HAZ and preventing the HAZ softening commonly observed with higher heat input welding processes.

### 3.3. Inhomogeneous Strain Behavior and Evolution of Strain Distribution during Tensile Testing

#### 3.3.1. Effect of Reinforcement on Tensile Strain Behavior in Pipeline Girth Welds

**Figure 5:**
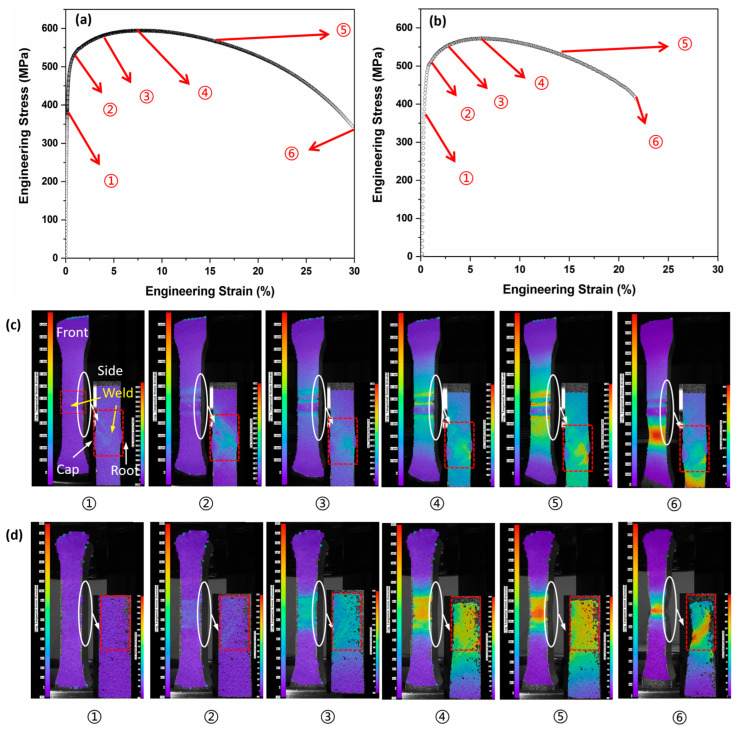
Stress-strain curve and the evolution of strain distribution of the SMAW-ed P1 sample during DIC-assisted tensile testing: (**a**,**b**) stress-strain curve of the SMAW-ed P1 sample with and without reinforcements, (**c**,**d**) evolution of strain distribution during the tensile testing with and without reinforcements. The red dashed rectangles in the figures indicate the welds regions from the side DIC view. The white circle is the location of the side view.

DIC-assisted tensile tests were conducted on the cross-weld of SMAW-ed P1 samples to evaluate the impact of reinforcement on the tensile strain behaviors of pipeline girth welds. In addition to the standard cross-weld samples featuring reinforcement (with specified dimensions of cap height of 1.5 mm and width of 20 mm), extra samples were prepared by removing the cap and root from the tensile specimens to create samples without reinforcement (zero reinforcement height). The stress-strain data depicted in Figure 5 and Table 3 illustrate that reinforcement enhances the mechanical properties of the girth weld. The yield strength and ultimate tensile strength of the reinforced girth weld are 525 MPa and 590 MPa, respectively, which are reduced to 502 MPa and 571 MPa after the removal of reinforcement, as shown in Table 3. The reinforced girth weld not only increases the strength but also improves the ductility. The total elongation of reinforced girth weld reaches 30%, which is significantly higher than 21% for that without reinforcement. The tensile properties of pure base metal (without girth weld) were also tested and are listed in Table 3 for reference. The strain hardening exponent, n, in the Hollomon model, is used to assess the strain hardening rate of the tensile tested samples [38]. The larger the value of n, the higher the strain hardening rate. It can be found that the reinforced samples present the highest hardening tendency.

Figure 5c,d illustrate the evolution of strain distribution and migration of maximum strain among inhomogeneous materials of base metal, weld, and HAZ as deformation increases in the girth weld with and without reinforcement. For clarity, six typical stages of strain distribution at different levels of deformation are shown, corresponding to (1) elastic deformation, (2) onset of yield point, (3) development of plastic deformation, (4) deformation at ultimate tensile strength, (5) necking, and (6) fracture, as labeled in Figure 5a,b. The front DIC image in Figure 5c,d captures the full deformation of the entire reduced section, as recorded by two front-facing cameras. The side DIC image in Figure 5c zooms in on the enlarged weld (including the cap, fill, and root), HAZ and base metal, as captured by two side cameras. The red dashed rectangles in the figures indicate the weld regions themselves.

The comparison between Figure 5c(①),d(①) demonstrates how the introduction of reinforcement alters the strain distribution at the elastic stage. Given that Young’s moduli of the base metal, weld metal, and HAZ are similar, no strain concentration is observed in the unreinforced girth weld, as depicted in Figure 5d(①). However, the addition of material at the cap and root in the reinforced girth weld introduces a geometric strengthening effect. As illustrated in the front view of Figure 5c(①), there are three areas of strain concentration. These areas correspond to the profile of the weld bead formed due to two cap passes shown in Figure 2a. The two outer areas of strain concentration are associated with the boundaries of the weld cap, where the HAZ is present. The central strain concentration area results from the intersection of two cap passes. This pattern highlights that the significant impact of geometrical strengthening is caused not only by reinforcement height but also reinforcement profile. The side view of Figure 5c(①) shows the extension of strain concentration from the weld toe to the weld root, associated with three strain concentration areas shown in the front view. The presence of these three strain concentration areas in the front view aids in identifying the weld region to analyze the subsequent DIC images.

At the onset of yielding, as illustrated in Figure 5c(②),d(②), the inhomogeneous material properties among the base metal, weld, and HAZ begin to influence the strain distribution, coupled with the geometric strengthening effect of the reinforcement. Figure 5d(②) shows a strain concentration in the softer regions, including the weld metal and HAZ upon yielding, which aligns well with the relatively low hardness of the weld and HAZ for unreinforced girth weld. In the case of the reinforced girth weld, as depicted in Figure 5c(②), the strain concentration is more pronounced in the HAZ and the intersection of two cap passes.

During the subsequent yielding stage, as depicted in Figure 5c(③), there is no significant change in strain distribution behaviors compared to that at the onset of yielding shown in Figure 5c(②). Clear strain concentration is still evident in the HAZ and at the intersection of two cap passes for the reinforced girth weld. The HAZ consistently exhibits the highest strain, with the strain concentration beginning to extend to the base metal adjacent to the HAZ. In contrast, when examining the strain distribution for the un-reinforced girth weld in Figure 5d(③), the strain continues to increase in the whole weld region, with a slightly higher concentration in the HAZ. This trend remains unchanged until the total stress reaches the ultimate tensile strength, as shown in Figure 5c(④),d(④).

When the total strain continues to increase, a certain part of the base metal begins to neck, resulting in a region with an extremely high strain, much greater than other areas. As illustrated in Figure 5d(⑤), the strain concentration remains within the weld metal and HAZ for the unreinforced sample. The maximum strain value in this region increases sharply, while the strain in other areas is still at a low level. However, for the sample with reinforcement shown in Figure 5c(⑤), the local strain at the HAZ increases slowly, while the local strain in a specific part of the base metal increases suddenly and exceeds that in the HAZ. The strain concentration migrates from the HAZ to the base metal. Figure 5c(⑥),d(⑥) show the subsequent strain evolution at fracture. It is obvious that the fracture occurs at the base metal for reinforced girth weld because of strain migration, while that remains at HAZ for the unreinforced sample.

**Figure 6:**
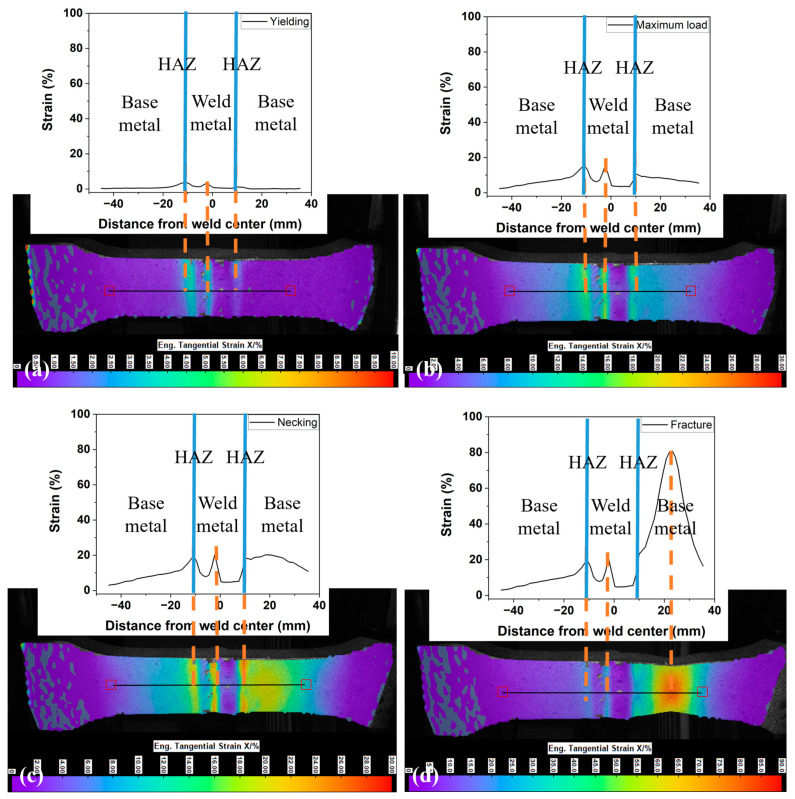
Strain evolution and migration on the central line of SMAW-ed P1 sample with reinforcement. (**a**) onset of yielding, (**b**) the maximum load, (**c**) necking, and (**d**) fracture.

The detailed DIC analysis of the front view provides insights into the maximum strain migration process from a macro perspective. Figure 6 illustrates the strain distribution along the central line of the SMAW-ed P1 sample with reinforcement at different stages. From yielding to the maximum load, as depicted in Figure 6a,b, strain concentration is observed at geometrically singular points, specifically the intersections of two weld passes, and in the HAZ characterized by lower hardness, while other areas exhibit smaller deformations. The highest strain is initially 4% at the onset of yielding and increases to 15% at the stage of maximum load (UTS point). Additionally, there is no migration of strain observed prior to reaching the maximum load. In Figure 6c, the strain concentration shifts to the base metal adjacent to the right HAZ, reaching a peak strain of 20%. This shift in strain concentration from previously higher-strain areas, such as the HAZ, to the base metal is referred to as maximum strain migration. Subsequently, as depicted in Figure 6d, the strain in the base metal adjacent to the right HAZ sharply increases to nearly 80%, leading to the fracture of the sample.

**Figure 7:**
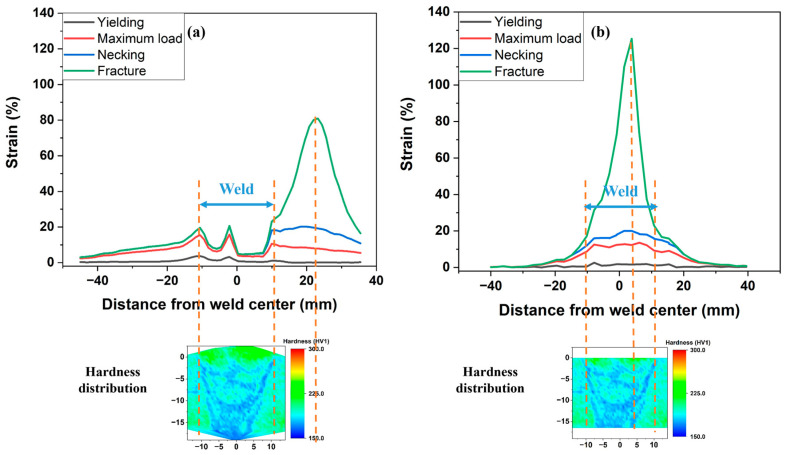
The comparison of strain migration between (**a**) SMAW-ed P1 sample with reinforcement, and (**b**) SMAW-ed P1 sample without reinforcement.

Figure 7 compares the strain migration behaviors between samples with and without reinforcement. In samples with reinforcement (Figure 7a), strain migration is evident, with maximum strain shifting from the weld and HAZ to the base metal as tensile deformation increases. This demonstrates the significant enhancement in girth weld strength due to reinforcement, effectively preventing failures at both the weld and HAZ, although hardness mapping indicated relatively lower hardness in this region. Conversely, in samples without reinforcement, strain continuously accumulates in the softer weld and HAZ throughout the tensile deformation process, as shown in Figure 7b. The remote strain in the base metal (−30~−40 mm from the weld center) distributes uniformly and remains almost unchanged after reaching the peak stress, with an average value of 0.4%. However, there is a notable increase in localized strain within the weld, achieving 13.5% under the maximum load. This localized strain then rapidly increases, ultimately leading to fracture in the weld rather than in the base metal. The design and construction Standard AS2885.1 [39], for elastic design, allows strains up to 0.5%. Our results show the girth weld without reinforcement will fail before reaching 0.5% allowable global strain. Considering no requirement for reinforcement in AS2885.2, the worst scenario will be the soft weld without reinforcement, the lack of reinforcement leads to minimal tolerance to displacement under displacement-controlled loadings, such as ground movement, as plastic deformation concentrates in the weld [25]. When the weld is strengthened by the reinforcement, the pipeline is capable of withstanding high displacement. Additionally, oil and gas pipelines are typically designed to operate within a temperature range of −20 °C to 120 °C. For pipelines in Arctic regions, the service temperature can be as low as −70 °C [40]. At low temperatures, the pipeline material can contract, leading to increased longitudinal tensile strain. Consequently, temperature fluctuations, particularly at low temperatures, significantly reduce the toughness of the pipeline steel and adversely affect the pipeline’s tensile strain capability. Therefore, the strengthening effect of the reinforcement is crucial in pipeline girth welds, particularly for undermatched welds.

#### 3.3.2. Mechanism of Strain Migration of Undermatched Girth Welds

**Figure 8:**
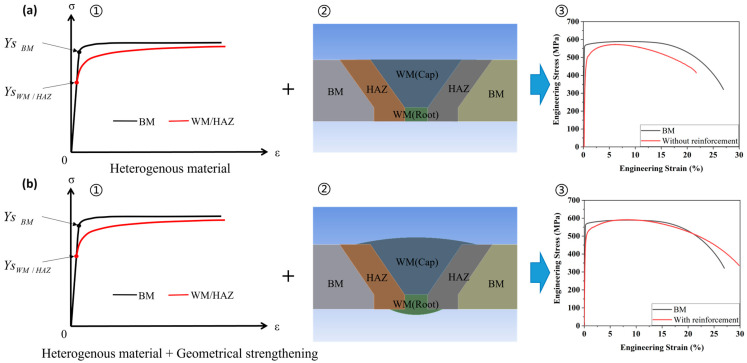
Mechanism of strain migration of undermatched girth welds. (**a**) without reinforcement and (**b**) with reinforcement, displaying (①) illustration of heterogeneous materials of BM, WM and HAZ; (②) illustration of reinforcement-induced geometry strengthening; (③) resultant actual cross-weld tensile deformation of SMAW-ed P1 sample.

The strain migration is a complex phenomenon, resulting from a combination of metallurgical and geometrical factors, as depicted in Figure 8. Metallurgically, the girth-welded pipe comprises heterogeneous materials including the base metal, weld metal, and HAZ. These diverse materials exhibit varying microstructures, mechanical properties, and stress-strain characteristics. As evidenced by the hardness maps presented in Figure 2a, the HAZ and weld metal exhibit lower hardness compared to the base metal, a characteristic indicative of undermatched girth welds. Geometrically, reinforcement plays a crucial role in strengthening the weld metal and HAZ by providing additional material at the weld toe and root. This added material enhances the structural integrity of the weld joint, contributing to improved mechanical properties and better resistance to stress and strain.

Figure 8a illustrates a girth weld without reinforcement, presenting an extreme scenario that remains within acceptable limits under current regulations. Strain concentration initially manifests at the yield point within the HAZ and weld metal, where the material strength is lower than that of the base metal. Although the HAZ and weld exhibit a higher strain hardening rate than that of BM (shown in Figure 8a(①)), stress-strain characteristics of the cross weld specimen comprised of HAZ, WM, and BM, as shown by the red curve in Figure 8a(③), consistently remain lower than that of a cross-weld specimen composed solely of base metal (pipe body), presented by the black curve in Figure 8a(③), due to the absence of reinforcement-induced strengthening. Consequently, the strain concentration persists in the softer HAZ and weld metal during subsequent necking, culminating in fracture predominantly within these regions. Considering the defects in weld and HAZ could further reduce the strength of girth weld, the undermatched girth weld without reinforcement scenario represents the undesirable weld performance in practice.

In Figure 8b, the dual effects of heterogeneous materials and reinforcement-induced geometrical strengthening contribute to distinctive strain evolution and migration properties. The reinforcement redistributes stress around the weld, enhancing the strength of the weld metal and HAZ [11,41]. The actual stress-strain characteristics of the cross-weld specimen with reinforcement, as shown by the red curve in Figure 8b(③), differ from those without reinforcement in Figure 8a(③). According to Table 3, the strain hardening exponents for welded samples, both with and without reinforcement, are comparable at 0.083 and 0.081, respectively. However, the reinforcement significantly increases the UTS of the reinforced samples to 590 MPa, exceeding that of the pipe body at 589 MPa, while the unreinforced sample is lower at 571 MPa. This reinforcement induces a notable transition in the stress-strain characteristics, as depicted in Figure 8b(③). Consequently, strain concentration originates in the HAZ and weld before reaching the UTS load, then migrates to the pipe body during necking, ultimately leading to fracture within the pipe body.

In summary, strain migration results from the combined effects of metallurgical and geometrical factors. For girth welded samples with reinforcement, at the onset of plastic deformation, the strengthening of the reinforcement is insufficient to offset the difference in yield strength, leading to strain concentration in the HAZ and weld where the yield strength is lower. As the load increases, the material undergoing plastic deformation experiences strain hardening. Since the strain hardening rate of the HAZ and weld is much higher than that of the base metal, the difference in strength between them decreases. Coupled with the strengthening effect of the reinforcement on the area around the weld, the base metal becomes the weakest region. Under the combined effects of strain hardening and reinforcement, strain concentration migrates from the HAZ and weld to the base metal, ultimately leading to fracture in the base metal. It demonstrates that the reinforcement effectively strengthens the girth weld and reduces the likelihood of failure in the sensitive regions (i.e., the weld metal and HAZ).

In the AS 2885.2 standard [12], there is no requirement for a minimum height of reinforcement. The worst scenario occurs where there is no reinforcement, as demonstrated in this research. Based on the above results and discussion, the influence of reinforcement on weld fracture behavior is vital and requires more attention. Therefore, to enhance the integrity of pipeline steels, the tensile test without reinforcement is recommended to qualify the welding procedure for girth welds.

#### 3.3.3. Effect of Welding Processes on Tensile Strain Evolution in Pipeline Girth Welds

**Figure 9:**
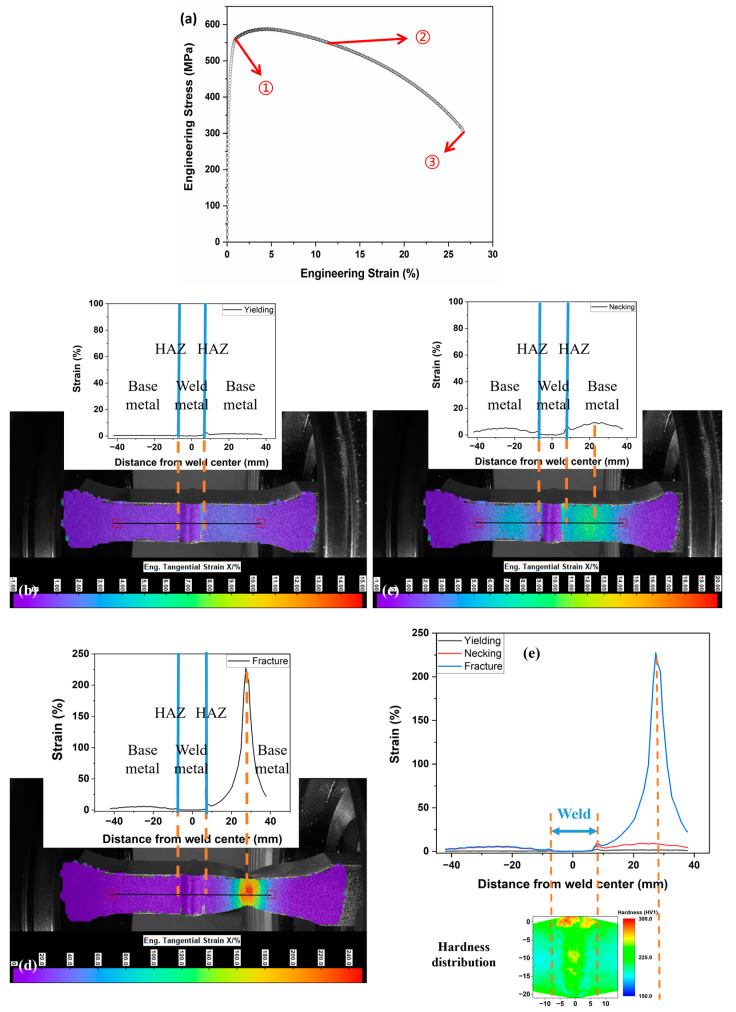
(**a**) Strain evolution and migration on the central line of GMAW-ed P2 sample with reinforcement (**b**) onset of yielding, (**c**) necking, (**d**) fracture; (**e**) different stage comparison.

To assess the impact of different welding processes on girth weld failures, we performed a comprehensive analysis of tensile strain behaviors in cross-weld samples created using SMAW and GMAW. For this analysis, we only focused on reinforced samples, as the unreinforced SMAW samples did not exhibit strain migration. The strain-stress curve, as depicted in Figure 9a, shows that the yield strength and UTS of the GMAW-ed P2 sample are 543 MPa and 589 MPa, respectively, close to the values of 525 MPa and 590 MPa for the SMAW-ed P1 sample. It is important to note that, as illustrated in the hardness mapping in Figure 2, GMAW results in a narrower weld and HAZ with higher hardness compared to the base metal, a characteristic indicative of overmatched girth welds. In contrast, SMAW-ed girth welds are typically undermatched.

Compared with the SMAW-ed P1 sample shown in Figure 6a, no strain concentration is observed in the HAZ upon yielding, as displayed in Figure 9b for the GMAW-ed P2 sample. In contrast, the strain distribution at the HAZ and weld metal is slightly lower than the rest of the base metal due to the overmatched girth weld. From yielding to necking, the reinforcement-induced geometric strengthening effect of the GMAW-ed P2 sample consistently makes the weld region stronger than the base metal, resulting in strain concentration occurring in the base metal, as shown in Figure 9c. After necking, the strain rapidly accumulates in a specific region of the base metal until fracture, while the weld region always retains a small amount of strain, as illustrated in Figure 9d.

Our research confirms that GMAW can effectively prevent HAZ softening and reduce strain concentration in HAZ. As shown in Figure 2, the HAZ of the SMAW-ed P1 sample is wider than that of the GMAW-ed P2 sample. Additionally, the hardness mappings reveal that softening occurs in the HAZ of the SMAW-ed P1 sample, whereas the GMAW-ed P2 sample exhibits no softening. This HAZ softening, induced by the welding process, significantly influences the tensile behavior of the samples. Consequently, for the undermatched SMAW-ed P1 sample, strain concentration initially occurs in the softer HAZ and weld, then migrates to the base metal due to strain hardening from metallurgical and geometrical strengthening effects, ultimately leading to fracture in the base metal. In contrast, the overmatched GMAW-ed P2 sample shows no strain migration. Throughout tensile deformation, the strain remains lower in the weld and higher in the adjacent base metal, where fracture ultimately occurs.

The difference in strain deformation performance between SMAW-ed and GMAW-ed girth welds can be attributed to variations in heat input between the two welding processes [42,43]. SMAW, characterized by its low welding speed and thermal efficiency, involves a higher heat input ranging from 0.9 to 1.8 kJ/mm for fill and cap passes, leading to a lower cooling rate and a wider HAZ. Conversely, GMAW features a lower heat input of 0.5 to 1.0 kJ/mm for these passes and a faster cooling rate, resulting in a narrower HAZ. Additionally, the microstructures of the HAZ differ between the two processes, as evidenced in Figure 3 and Figure 4. For GMAW, the rapid cooling conditions lead to the formation of smaller bainitic laths and dispersed precipitation of micro-alloyed carbon and nitride compounds, which enhance the mechanical properties [44,45]. Moreover, the faster cooling rate contributes to a finer grain size in the HAZ, thereby improving strength and hardness [46,47].

## 4. Conclusions

The current study utilizes 3D Digital Image Correlation to analyze the inhomogeneous strain behavior and the evolution of strain distribution during tensile testing of high-strength, thick-walled X65 pipelines. This approach provides critical insights into strain migration and fracture mechanisms specific to in-service pipeline girth welds. The effects of various welding processes, as well as the presence or absence of reinforcements, are investigated in detail. The main findings are summarized below:

For the tested SMAW-ed pipe, both the HAZ and weld metal exhibit lower hardness compared to the base metal, which is characteristic as undermatched girth welds. In contrast, GMAW results in a narrower weld and HAZ with higher hardness than the base material, indicating overmatched girth welds. No HAZ softening occurs in the GMAW-ed pipe due to the low heat input.

Strain migration results from a combination of metallurgical and geometrical factors. Under tensile deformation, strain concentration occurs in the softer HAZ and weld of the reinforced SMAW-ed sample, then migrates to the base metal, ultimately leading to fracture in the base metal. Conversely, the GMAW-ed sample exhibits no strain migration; the strain remains lower in the weld and higher in the adjacent base metal, where fracture ultimately occurs.

Reinforcement significantly influences the tensile properties by redistributing stress around the weld. It enhances the tensile strength of girth welds and effectively prevents failure in the weld region. Adequate reinforcement is crucial, as it reduces the likelihood of failure in sensitive areas such as the weld metal and HAZ, especially for SMAW-ed pipes. It is worth noting that the AS 2885.2 standard does not require minimum reinforcement. Therefore, it is recommended to conduct tensile tests without reinforcement during welding procedure qualification.

It is recommended to further investigate the influence of reinforcement geometry, including height and width, on the longitudinal tensile properties of SMAW-ed pipelines. Hence, welding standards could specify the minimum height for reinforcement.

## Figures and Tables

**Table 1:** Chemical composition of pipeline steel in this study (wt%).

	C	Si	Mn	P	S	Cu	Ni
P1	0.040	0.18	1.15	0.0061	0.0009	0.19	0.10
P2	0.041	0.24	1.24	0.0061	0.0011	0.23	0.18
**Cr**	**Mo**	**V**	**Nb**	**Al**	**Ti**	**B**	**N**
0.17	0.059	0.0014	0.039	0.035	0.0133	0.0002	0.0034
0.16	0.057	0.0414	0.042	0.027	0.0061	0.0002	0.0040

**Table 2:** Welding procedure of pipeline girth welds.

Base Metal	Wall Thickness	Welding Process	Welding Consumables		Heat Input Range (kJ/mm)	
			Root	Fill & Cap	Root	Fill & Cap
P1	17.5 mm	SMAW/GMAW	ER70S-G	E8018-G	0.5~0.8	0.9~1.8
P2	19.8 mm	GMAW	ER70S-G	ER70S-G	0.4~0.6	0.5~1.0

**Table 3:** Mechanical properties of P1 in different conditions.

	Yield Strength/MPa	Ultimate Tensile Strength/MPa	Elongation/%	Stain Hardening Rate
With reinforcement	525	590	30	0.083
Without reinforcement	502	571	21	0.081
Base metal	562	589	27	0.055

## Data Availability

The data presented in this study are available on request from the corresponding author. The data are not publicly available due to privacy.

## List of Abbreviations

**AF**	Acicular ferrite
**BF**	Bainitic ferrite
BM	Base metal
CGHAZ	Coarse-grain heat-affected zone
DIC	Digital image correlation
FGHAZ	Fine-grain heat-affected zone
GB	Granular bainite
GF	Grain boundary ferrite
GMAW	Gas metal arc welding
HAZ	Heat-affected zone
ICCGHAZ	Inter-critical reheated coarse-grain heat-affected zone
ICHAZ	Inter-critical heat-affected zone
M/A	Martensite–austenite constituent
P	Pearlite
PF	Polygonal ferrite
SCHAZ	Sub-critical heat-affected zone
SMAW	Shielded metal arc welding
UTS	Ultimate tensile strength
